# New *Arthrobotrys* Nematode-Trapping Species (Orbiliaceae) from Terrestrial Soils and Freshwater Sediments in China

**DOI:** 10.3390/jof8070671

**Published:** 2022-06-26

**Authors:** Fa Zhang, Saranyaphat Boonmee, Jayarama D. Bhat, Wen Xiao, Xiao-Yan Yang

**Affiliations:** 1 Institute of Eastern-Himalaya Biodiversity Research, Dali University, Dali 671003, China; zhangf@eastern-himalaya.cn (F.Z.); xiaow@eastern-himalaya.cn (W.X.); 2 Center of Excellence in Fungal Research, Mae Fah Luang University, Chiang Rai 57100, Thailand; saranyaphat.boo@mfu.ac.th; 3 School of Science, Mae Fah Luang University, Chiang Rai 57100, Thailand; 4 Department of Botany, Goa University, Taleigao 403206, India; bhatdj@gmail.com; 5 Key Laboratory of Yunnan State Education Department on Er’hai Lake Basin Protection and the Sustainable Development Research, Dali University, Dali 671003, China; 6 Yunling Back-and-White Snub-Nosed Monkey Observation and Research Station of Yunnan Province, Dali 671003, China

**Keywords:** 6 new taxa, molecular phylogeny, morphological, nematode-trapping hyphomycetes, taxonomy

## Abstract

*Arthrobotrys* is the most complex genus of Orbiliaceae nematode-trapping fungi. Its members are widely distributed in various habitats worldwide due to their unique nematode-trapping survival strategies. During a survey of nematophagous fungi in Yunnan Province, China, twelve taxa were isolated from terrestrial soil and freshwater sediment habitats and were identified as six new species in *Arthrobotrys* based on evidence from morphological and multigene (ITS, TEF, and RPB2) phylogenetic analyses. These new species i.e., *Arthrobotrys eryuanensis*, *A. jinpingensis*, *A. lanpingensis*, *A. luquanensis*, *A. shuifuensis*, and *A. zhaoyangensis* are named in recognition of their places of origin. Morphological descriptions, illustrations, taxonomic notes, and a multilocus phylogenetic analysis are provided for all new taxa. In addition, a key to known species in *Arthrobotrys* is provided, and the inadequacies in the taxonomic study of nematode-trapping fungi are also discussed.

## 1. Introduction

Nematophagous fungi are a group of fungi that parasitize, capture, and poison nematodes and important balancing agents of the nematode population in nature [1,2,3]. They were divided into different groups according to their mode of action on nematodes: (1) nematode-trapping fungi capture nematodes with specialized hypha structure, (2) endoparasitic fungi infect nematodes with spores, (3) egg parasitic fungi invade nematode eggs and females with hypha tips, and (4) toxin-producing fungi produce toxins that paralyze and kill nematodes [3,4,5]. Among these, nematode-trapping fungi have been the focus of related studies due to their highly specialized, sophisticated, and diverse trapping structures. Since Corda described the first nematode-trapping species (*Arthrobotrys superba* Corda) [6], more than 120 species have been discovered in Zygomycota (Zoopagaceae), Basidiomycota (*Nematoctonus*), and Ascomycota (Orbiliomycetes) over the past 180 years [5,7,8]. Nematode-trapping fungi in Zygomycota (Zoopagaceae) are poorly understood due to their immature isolation and culture methods [8,9]. All nematode-trapping fungi in Basidiomycota catch nematodes with adhesive knobs or adhesive spores, and all of them belong to *Nematoctonus* [8,10,11,12]. All nematode-trapping fungi in the Ascomycota belong to Orbiliaceae (the only family of Orbiliomycetes), accounting for more than 80% of all nematode-trapping fungi, which is a typical monophyletic group. They capture nematodes by producing constricting rings, adhesive networks, adhesive branches, adhesive knobs, and non-constricting rings [4,13].

Orbiliaceae nematode-trapping fungi have become the focus of studies on carnivorous fungi and also is a focus group of fungal evolutionists due to their unique survival strategies, diverse and complex trapping structures, abundant species, and relatively mature research methods [13,14,15,16]. At present, 103 species have been discovered [4,17,18,19]. The history of its taxonomic research can be roughly divided into two periods: (1) from 1839 to about 1995, 26 genera were established to accommodate these species based on the morphological characteristics of conidia and conidiophores. With the subsequent discovery of more and more species, systematic comparative morphological studies were carried out, and the idea of dividing Orbiliaceae nematode-trapping fungi into *Arthrobotrys*, *Dactylella*, and *Monacrosporium* was proposed and widely accepted [19]. (2) Since 1995, with the development of molecular biology techniques, molecular phylogenetic studies based on DNA sequences, restriction fragment length polymorphism (RFLP), and random amplified polymorphic DNA (RAPD) indicate that species with the same trapping structure have closer phylogenetic relationships. Additionally, the idea that the types of trapping devices are more informative than conidia and conidiophores for the division of genera among Orbiliaceae nematode-trapping fungi was proposed. All Orbiliaceae nematode-trapping fungi are also classified into *Arthrobotrys*, *Dactylellina*, or *Drechserella* according to their types of trapping structure [4,8,14].

*Arthrobotrys* is the largest genus among Orbiliaceae nematode-trapping fungi. At present, 118 records of *Arthrobotrys* are listed in the Species Fungorum (http://www.speciesfungorum.org; (accessed on 6 March 2022)), which represent 59 accepted species [4,5,8,13,19]. It was established by Corda (1839), with *A. superba* Corda as the type species. These taxa are characterized by regularly 1-septate conidia growing on the nodes or short denticles of conidiophores [6]. At the time of its establishment, this genus was known for saprobic taxa [6,20]. Zopf (1888) provided a detailed description of a unique phenomenon in which *A*. *oligospora* produces adhesive networks to capture nematodes and clarified the relationship between *Arthrobotrys* and nematodes [21]. In the following decades, due to the limitations of the available research techniques, the understanding of this group remained relatively poor. It was not until Drechsler and Duddington (1933) improved the isolation method that an increasing number of species were discovered [22,23,24,25,26,27,28,29,30,31,32,33]. Because scholars attached different levels of importance to different morphological features, these species were parked in several genera such as *Didymozoophaga*, *Anilosporium*, and *Drechsleromyces* [34,35,36]. Subsequently, scholars redefined the characteristics of the genus *Arthrobotrys* by systematic comparative morphological studies as follows: branched or simple conidiophores; obovoid, elliptic, pyriform, 0–3-septate conidia, growing asynchronously on the nodes or on short denticles of conidiophores; and including species that capture nematodes with adhesive networks, constricting rings, and adhesive knobs [36,37,38,39,40,41,42]. Subsequently, modern molecular biology techniques have been used to explore the taxonomy of Orbiliaceae nematode-trapping fungi and indicate that species with adhesive networks usually have similar molecular characteristics. Therefore, the main characteristic of *Arthrobotrys* was correspondingly changed to producing an adhesive network to capture nematodes [4,8,14,15]. In addition, *Arthrobotrys* is the most widely distributed nematode-trapping fungi and the dominant group in most habitats. They mainly occur in the soil or sediment of various ecosystems such as farmland, forests, mangroves, and freshwater, and they are also recorded in hot springs, animal waste, and tree trunks [3,17,18,31,34,43,44,45,46,47,48]. Most *Arthrobotrys* species have strong saprophytic and reproductive capacity and can quickly colonize in soil [3,4,19], so they are ideal materials for the development of parasitic nematode biocontrol agents. At the same time, they are also a good group for the evolutionary studies of nematode-trapping fungi within the genus because of the abundant species and obvious morphological differentiation of conidia and conidiophores [4,19]. The six new species described in this study enhance the diversity of nematode-trapping fungi, provide more materials for the biological control of parasitic nematodes, and add precious research objects for evolutionary studies of nematode-trapping fungi.

## 2. Materials and Methods

### 2.1. Sampling, Fungal Isolation and Morphological Observation

The strains included in this study were isolated from terrestrial soil and freshwater sediment collected in Yunnan Province, China. Terrestrial soil samples were collected from 0–10 cm depth using a 35 mm-diameter soil borer after removing fallen leaves from the soil surface [49,50,51]. Freshwater sediment samples were removed from the water with a Peterson bottom sampler (HL-CN, Wuhan Hengling Technology Company, Limited, Wuhan, China). The samples were placed into a zip-lock bag, and relevant site information were recorded. The samples were stored at 4 °C until processing.

Samples of 1–2 g of soil or sediment were spread on the surface of cornmeal agar (CMA) plates with sterile toothpicks. Approximately 5000 nematodes (*Panagrellus redivivus* Goodey, free-living nematodes) were added as bait to promote the germination of the nematode-trapping fungi [4,32,52,53]. The plates were incubated at 26 °C for three weeks and then observed under a stereomicroscope; the spores of nematode-trapping fungi were transferred to fresh CMA plates using a sterile needle. This step was repeated until a pure culture was obtained [4,5]. 

The pure cultures were transferred to fresh CMA plates with observation well (a square slot 2 × 2 cm created by removing agar in each plate) using a sterile needle and incubated at 26 °C until the mycelium spread beyond the well. Approximately 1000 living nematodes were placed in the well to induce the formation of the trapping device [4,5]. The types of trapping devices were checked using a stereomicroscope. All micromorphological features were photographed and measured with an Olympus BX53 microscope (Olympus Corporation, Tokyo, Japan).

### 2.2. DNA Extraction, PCR Amplification and Sequencing

Total genomic DNA was extracted from mycelium grown on potato dextrose agar (PDA) plates using a rapid fungal genomic DNA isolation kit (Sangon Biotech Company, Limited, Shanghai, China). The ITS, TEF, and RPB2 regions were amplified with the primer pairs ITS4-ITS5 [54], 526F-1567R [55], and 6F-7R [56], respectively. The PCR amplification was performed as follows: 4 min of pre-denaturation at 94 °C; followed by 35 cycles of 45 s denaturation at 94 °C; 1 min of annealing at 52 °C (ITS), 55 °C (TEF), or 54 °C (RPB2), and 1.5–2 min of extension at 72 °C; with a final extension of 10 min at 72 °C. The PCR products were purified with a DiaSpin PCR Product Purification Kit (Sangon Biotech Company, Limited, Shanghai, China). The purified PCR products of the ITS and RPB2 regions were sequenced in the forward and reverse directions using PCR primers, and the primer pair 247F-609R [57] was used to sequence the TEF genes (BioSune Biotech Company, Limited, Shanghai, China). SeqMan v. 7.0 (DNASTAR, Madison, WI, USA) [58] was used to check, edit, and assemble the sequences. The sequences generated in this study were deposited in the GenBank database at the National Center for Biotechnology Information (NCBI; https://www.ncbi.nlm.nih.gov/, accessed on 26 February 2022), and the accession numbers are listed in Table 1.

### 2.3. Phylogenetic Analysis

The sequences generated in this study were compared against the NCBI GenBank database using BLASTn (https://blast.ncbi.nlm.nih.gov/, accessed on 11 February 2022). The BLASTn search results and the morphological features of these six species indicated that they belong to the genus *Arthrobotrys.* This genus was searched in the Species Fungorum (http://www.speciesfungorum.org, accessed on 13 February 2022), and all relevant records were checked individually according to the relevant documents to ensure that all *Arthrobotrys* taxa were considered in this study [4,5,8,13,19]. All reliable ITS, TEF, and RPB2 sequences of *Arthrobotrys* taxa were downloaded from GenBank database (Table 1). Three genes were aligned using the online program MAFFT v.7 (http://mafft.cbrc.jp/alignment/server/, accessed on 15 February 2022) [67] and manually adjusted using BioEdit v7.2.3 [68]; they were then linked with MEGA6.0 [69]. *Vermispora fusarina* YXJ13-5 and *Dactylaria higginsii* CBS 121934 were selected as outgroups. Phylogenetic trees were inferred via maximum likelihood (ML), maximum parsimony (MP), and Bayesian inference (BI) analyses.

The SYM+I+G, GTR+I+G, and GTR+I+G models were selected via jModelTest v2.1.10 [70] as the best-fit optimal substitution models for ITS, TEF, and RPB2, respectively, for maximum likelihood (ML) and Bayesian inference (BI) analysis.

Maximum likelihood (ML) analysis was implemented using IQ-Tree v1.6.5 [71]. The dataset was partitioned, and each gene was analysed with the corresponding model. The statistical bootstrap support values (BS) were computed using rapid bootstrapping with 1000 replicates [72].

PAUP 4. a168 on XSEDE [73] in the CIPRES Science Gateway v. 3.3 web resource was used to generate the maximum parsimony (MP) analysis. Trees were inferred using the heuristic search option with TBR branch swapping and 1000 random sequence additions. Max-trees were set up at 5000 and no-increase. Clade stability was assessed via a bootstrap analysis with 1000 replicates [72]. Tree length (TL), consistency index (CI), retention index (RI), rescaled consistency index (RC), and homoplasy index (HI) values were calculated for all trees generated under different optimality criteria. All of the above parameters were edited into the PAUP block in the NEX file.

Bayesian inference (BI) analysis was conducted with MrBayes v. 3.2.6. [74]. The multiple sequence alignment file was converted into a MrBayes-compatible NEXUS file using FastaConvert [75]. The dataset was partitioned, and the optimal substitution models of each gene were equivalently replaced to conform to the setting of MrBayes. Six simultaneous Markov chains were run for 10,000,000 generations, and trees were sampled every 100 generations. The first 25% of the trees were discarded, and the remaining trees were used to calculate the posterior probabilities (PP) in the majority rule consensus tree. All of the above parameters were edited in the MrBayes block in the NEX file.

The tree was visualized with FigTree v1.3.1 [76]. The backbone tree was edited and reorganized using Microsoft PowerPoint (2013) and Adobe Photoshop CS6 software (Adobe Systems, San Jose, CA, USA).

## 3. Results

### 3.1. Phylogenetic Analysis

**Figure 1:**
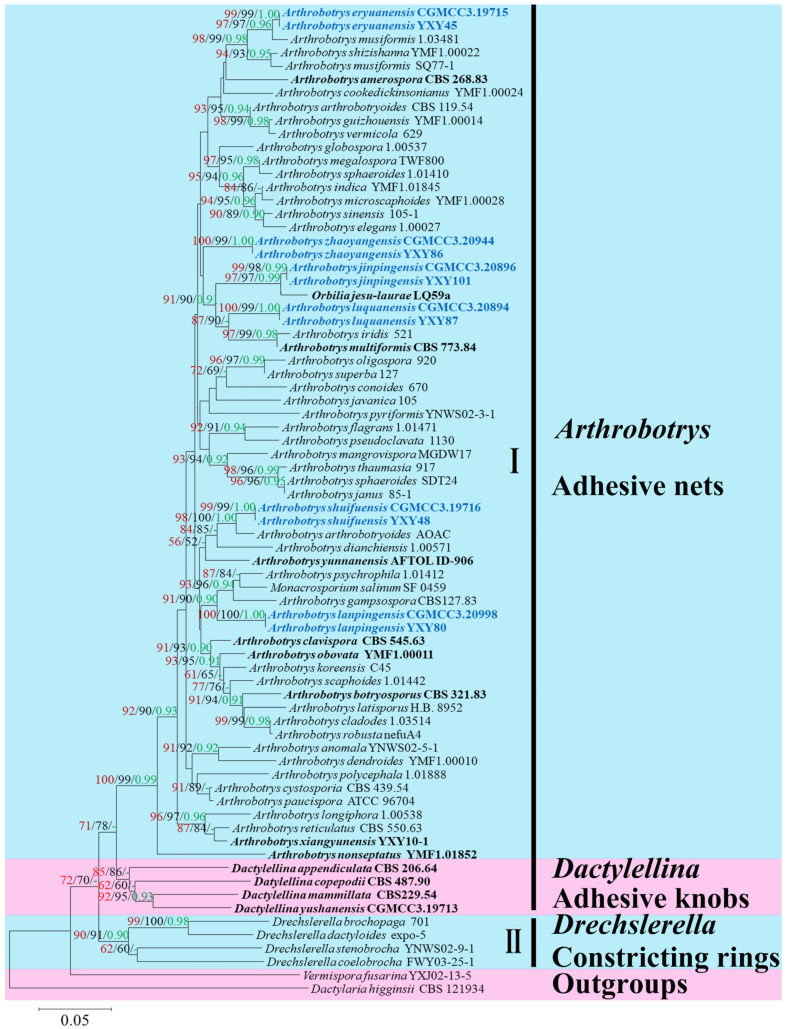
Maximum likelihood tree based on a combined ITS, TEF, and RPB2 sequence from 65 species of Orbiliaceae nematode-trapping fungi. Bootstrap support values for maximum likelihood (red) and maximum parsimony (black) greater than 50% and Bayesian posterior probabilities values (green) greater than 0.90 are indicated above the nodes. The new isolates are in blue; type strains are in bold. The tree is rooted by *Vermispora fusarina* YXJ13-5 and *Dactylaria higginsii* CBS 121934.

A total of 118 *Arthrobotry* related taxa were listed in the Species Fungorum (http://www.speciesfungorum.org/ (accessed on 6 March 2022)), representing 59 valid *Arthrobotrys* species. Among them, 51 species had confirmed molecular data. Therefore, the combined ITS, TEF, and RPB2 alignment dataset contained 64 *Arthrobotrys* isolates representing 57 *Arthrobotrys* species (plus our 12 isolates and 6 new species) and other related species in Orbiliaceae (*Dactylellina*: 4 species and *Drechslerella*: 4 species). The final dataset comprised 1918 characters (551 for ITS, 547 for TEF, and 820 for RPB2), among which 872 bp were constant, 1004 bp were variable, and 748 bp were parsimony informative. The maximum likelihood analysis of a best-scoring tree was performed with a final ML optimization likelihood value of −6304.618465. Within the MP analysis, a strict consensus MP tree was obtained from the three most equally parsimonious trees (TL = 3443, CI = 0.546, RI = 0.510, RC = 0.298, HI = 0.419). For the Bayesian analysis (BI), the consensus tree was calculated with the remaining 75% of trees, and the Bayesian posterior probabilities were evaluated with a final average standard deviation of the split frequency of 0.009254. Although the trees inferred by ML, MP, and BI showed slightly different topologies in some clusters, all trees showed that all six species clustered together with known *Arthrobotrys* species, with distinct divergence from other species. The best-scoring ML tree was selected for presentation (Figure 1).

The phylogram inferred from the ITS+TEF+RPB2 dataset showed these six species clustered in *Arthrobotrys*. Among these species, *Arthrobotrys eryuanensis* clustered together with *A. musiformis* and *A. shizishanna* with 98% MPBS, 99% MLBS, and 0.98 BYPP support. *Arthrobotrys jinpingensis* and *A. shuifuensis* were sisters to *Orbilia jesu-laurae* and *A. arthrobotryoides*, respectively, with high support values (95% MPBS, 95%MLBS, 0.95 BYPP). *Arthrobotrys luquanensis* formed a basal lineage with *A. iridis* and *A. multiformis* with 87% MPBS and 90% MLBS support. *Arthrobotrys lanpingensis* clustered together with *A. psychrophila*, *A. salinum*, and *A. gampsospora* with 91% MPBS, 90% MLBS, and 0.90 BYPP support. The phylogenetic position of *Arthrobotrys zhaoyangensis* was uncertain, but this species showed significant divergence from known species.

### 3.2. Taxonomy

**Figure 2:**
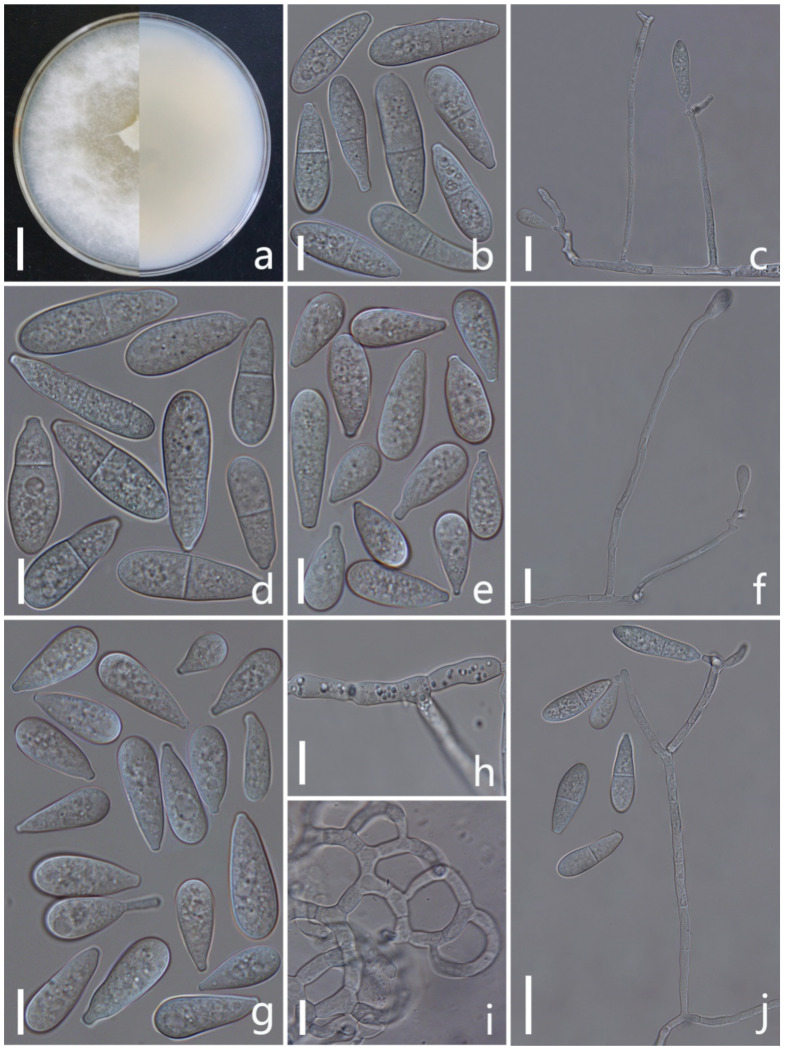
*Arthrobotrys eryuanensis* (CGMCC3.19715). (**a**) Colony. (**b**,**d**) Macroconidia. (**e**,**g**) Microconidia. (**c**,**f**,**j**) Conidiophores. (**h**) Chlamydospores. (**i**) Trapping device: adhesive networks. Scale bars: (**a**) = 1 cm, (**b**,**d**,**e**,**g**–**i**) = 10 μm, (**c**,**f**,**j**) = 20 μm.

*Arthrobotrys eryuanensis* F. Zhang & X.Y. Yang sp. nov. (Figure 2).

Index Fungorum number: IF556938; Facesoffungi number: FoF 10760

Etymology: The species name “eryuanensis” refers to the name of the sample collection site: Eryuan County, Dali City, Yunnan Province, China.

Material examined: CHINA, Yunnan Province, Dali City, Eryuan County, Xihu Lake, 26°9′8.77″ N, 99°57′17.03″ E, from freshwater sediment, 20 June 2014, F. Zhang. Holotype CGMCC3.19715, preserved in the China General Microbiological Culture Collection Center. Ex-type culture DLUCC 14-1, preserved in the Dali University Culture Collection.

*Colonies* on PDA white, cottony, growing rapidly, reaching 50 mm diameter after 7 days in the incubator at 26 °C. *Mycelium* partly superficial, partly immersed, composed of septate, branched, smooth hyphae. *Conidiophores* 110–308 µm (x = 213.5 µm, *n* = 50) long, 2.5–4.5 µm (x = 3.2 µm, *n* = 50) wide at base, gradually tapering upwards to apex, 1.5–3 µm (x = 2.2 µm, *n* = 50) wide at apex, erect, septate, branched, hyaline, producing 2–10 short polyblastic denticles at apex, with each denticle bearing a single holoblastic conidium. *Conidia* two types: *Macroconidia* 18–44.5 × 5–11.5 µm (x = 28.4 × 8.7 μm, *n* = 50), clavate to elongate pyriform, some slightly curved, wider rounded at apex, narrower towards the lower with truncate at base, 1-septate, septum median to submedian, hyaline, guttulate. *Microconidia* 7.5–28 × 4–11 µm (x = 17.6 × 8.6 μm, *n* = 50), subglobose to clavate, obovoid, wider rounded at apex, truncate at papillate bulged base, aseptate, hyaline, guttulate. *Chlamydospores* 7–18.5 × 3.5–8 µm (x = 10.7 × 5.8 μm, *n* = 50), cylindrical, hyaline, in chains when present, sometimes guttulate, slightly verruculose-walled. Captures nematodes with adhesive networks.

Additional specimen examined: CHINA, Yunnan Province, Dali City, Eryuan County, Xihu Lake, 26°9′8.77″ N, 99°57′17.03″ E, from freshwater sediment, 20 June 2014, F. Zhang. Living culture YXY45.

Notes: Phylogenetically, *Arthrobotrys eryuanensis* clusters together with *A. shizishanna* and *A. musiformis* with high support values (98% MLBS, 99% MPBS, 0.99 BYPP). *A. eryuanensis* was 6.7% (39/586 bp) and 5.3% (26/486 bp) different from *A. shizishanna* and *A. musiformis* in ITS sequence. Morphologically, *A. eryuanensis* can be easily distinguished from *A. shizishanna* in shape, size, septation, and numbers of conidia and conidiophores [77]. It is more similar to *A. musiformis* in the morphology of its macroconidia [4,19]. Their differences are as follows: (1) *A. musiformis* produces one type of conidia, most of which are curved, while *A. eryuanensis* produces two types of conidia. Macroconidia is 1-septate, partly curved and partly symmetrical, and microconidia is aseptate and truncate at the base with a papillate bulge. (2) The conidiophores of *A. musiformis* are unbranched, while most of those in *A. eryuanensis* are branched.

**Figure 3:**
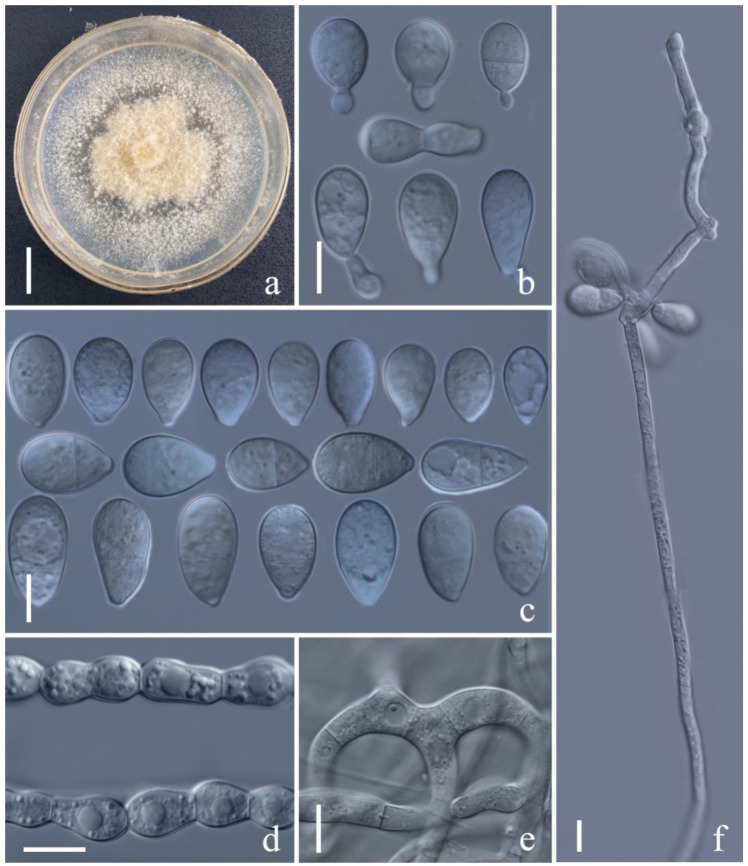
*Arthrobotrys jinpingensis* (CGMCC3.20896). (**a**) Colony. (**b**,**c**) Conidia. (**d**) Chlamydospores. (**e**) Trapping device: adhesive networks. (**f**) Conidiophore. Scale bars: (**a**) = 1 cm, (**b**–**f**) = 10 µm.

*Arthrobotrys jinpingensis* F. Zhang & X.Y. Yang sp. nov. (Figure 3).

Index Fungorum number: IF 556018; Facesoffungi number: FoF 10761.

Etymology: The species name “jinpingensis” refers to the name of the sample collection site: Jinping County, Gejiu City, Yunnan Province, China.

Material examined: CHINA, Yunnan Province, Gejiu City, Jinping County, 23°4′54.80″ N, 103°12′40.80″ E, from terrestrial soil, 19 April 2017, F. Zhang. Holotype CGMCC3.20896, preserved in the China General Microbiological Culture Collection Center. Ex-type culture DLUCC 21-1, preserved in the Dali University Culture Collection.

*Colonies* on PDA white, cottony, growing rapidly, reaching 60 mm diameter after 10 days in the incubator at 27 °C. *Mycelium* partly superficial, partly immersed, composed of septate, branched, smooth hyphae. *Conidiophores* 225–509 µm (x = 348.2 µm, *n* = 50) long, 3–8.5 µm (x = 4.9 µm, *n* = 50) wide at base, gradually tapering upwards to apex, 1.5–3 µm (x = 2.1 µm, *n* = 50) wide at apex, erect, septate, unbranched, hyaline, producing several separate nodes by the repeated elongation of conidiophores, with each node bearing 2–11 polyblastic conidia. *Conidia* 11–26.5 × 6.5–14.5 µm (x = 18.6 × 10.8 μm, *n* = 50), subglobose, oval to obovoid, obpyriform, wider rounded at apex, narrow towards with truncate at base, sometimes with a bud-like projection at base, 0 or 1-septate, hyaline, rough to smooth-walled. *Chlamydospores* 7–18.5 × 5.5–9.5 µm (x = 13.3 × 7.4 μm, *n* = 50), cylindrical, ellipsoidal, in chains, hyaline, guttulate, rough-walled. Captures nematodes with adhesive networks.

Additional specimen examined: CHINA, Yunnan Province, Gejiu City, Jinping County, 23°4′54.80″ N, 103°12′40.80″ E, from terrestrial soil, 19 April 2017, F. Zhang. Living culture YXY101.

Notes: Phylogenetically, *Arthrobotrys jinpingensis* forms a sister lineage to *Orbilia jesu-laurae* with 97% MLBS, 97% MPBS, 0.99 BYPP support. There is 2.5% (15/600 bp) difference in their ITS sequences. However, the conidiophores of *A. jinpingensis*is are unbranched, producing several separate nodes by repeated elongation, while the conidiophores of *O. jesu-laurae* are branched and produce only one node at apex. In addition, some conidia of *A. jinpingensis* have a bud-like projection at base, while the conidia of *O. jesu-laurae* do not [76].

**Figure 4:**
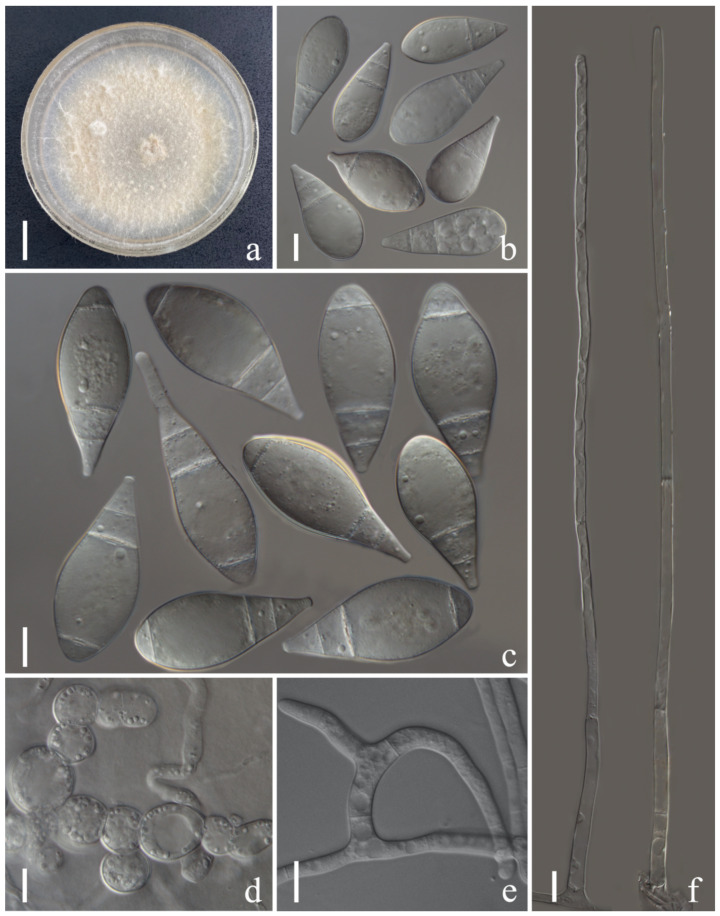
*Arthrobotrys lanpingensis* (CGMCC3.20998). (**a**) Colony. (**b**,**c**) Conidia. (**d**) Chlamydospores. (**e**) Trapping device: adhesive networks. (**f**) Conidiophores. Scale bars: (**a**) = 1 cm, (**b**–**f**) = 10 µm.

*Arthrobotrys lanpingensis* F. Zhang & X.Y. Yang sp. nov. (Figure 4).

Index Fungorum number: IF559021; Facesoffungi number: FoF 10762.

Etymology: The species name “lanpingensis” refers to the name of the sample collection site: Lanping County, Nujiang City, Yunnan Province, China.

Material examined: CHINA, Yunnan Province, Nujiang City, Lanping County, 26°22′13.50″ N, 99°23′0.20″ E, from freshwater sediment, 16 May 2015, F. Zhang. Holotype CGMCC3.20998, preserved in the China General Microbiological Culture Collection Center. Ex-type culture DLUCC 18-1, preserved in the Dali University Culture Collection.

*Colonies* on PDA white, cottony, growing rapidly, reaching 50 mm diameter after 10 days in the incubator at 27 °C. *Mycelium* partly superficial, partly immersed, composed of septate, branched, smooth hyphae. *Conidiophores* 241–503 µm (x = 307.5 µm, *n* = 50) long, 3.5–7 µm (x = 4.7 µm, *n* = 50) wide at base, gradually tapering upwards to apex, 2–3.5 µm (x = 2.4 µm, *n* = 50) wide at apex, erect, septate, unbranched, hyaline, bearing a single holoblastic conidium at apex. *Conidia* 31–55 × 13.5–24.5 µm (x = 45.4 × 19.7 μm, *n* = 50), obovoid, cuneiform to slightly pyriform, upper cell wider than lower cell, apex rounded, widest at median cell, tapering towards the narrow and subacute with truncate base, 1-septate when immature, becoming 3-septate at maturity (2 at base and 1 at apex), hyaline, minutely guttulate, smooth-walled. *Chlamydospores* 8–27 × 8–25 µm (x = 17.4 × 14.5 μm, *n* = 50), globose to subglobose or ellipsoidal, growing in chains, hyaline, guttulate, rough-walled. Capturing nematodes with adhesive networks.

Additional specimen examined: CHINA, Yunnan Province, Nujiang City, Lanping County, 26°22′13.50″ N, 99°23′0.20″ E, from freshwater sediment, 16 May 2015, F. Zhang. Living culture YXY80.

Notes: Phylogenetically, *Arthrobotrys lanpingensis* formed a sister lineage to *A. psychrophila*, *A. salinum* and *A. gampsospora* with 91% MLBS, 90% MPBS, and 0.90 BYPP support. *A. lanpingensis* was 9.3% (56/602 bp), 6.4% (32/503 bp), and 8.7% (50/576 bp) different from *A. gampsospora*, *A. psychrophile*, and *A. salinum* in ITS sequences, respectively. Morphologically, *A. lanpingensis* is most similar to *A. guizhouensis* in their subfusiform conidia. However, *A. guizhouensis* produces two types of conidia, while *A. lanpingensis* produces only one type of conidia. In addition, most conidia of *A. lanpingensis* are 3-septate, whereas the conidia of *A. guizhouense* are 2-septate, and the conidia of *A. lanpingensis* are significantly smaller than those of *A. guizhouensis* [*A. lanpingensis*, 31.1–55.2 (45.4) × 13.5–24.3 (19.7) µm versus *A. guizhouensis*, 30.5–71.5 (52.7) × 18.5–28.5 (23.9) µm] [4,19].

**Figure 5:**
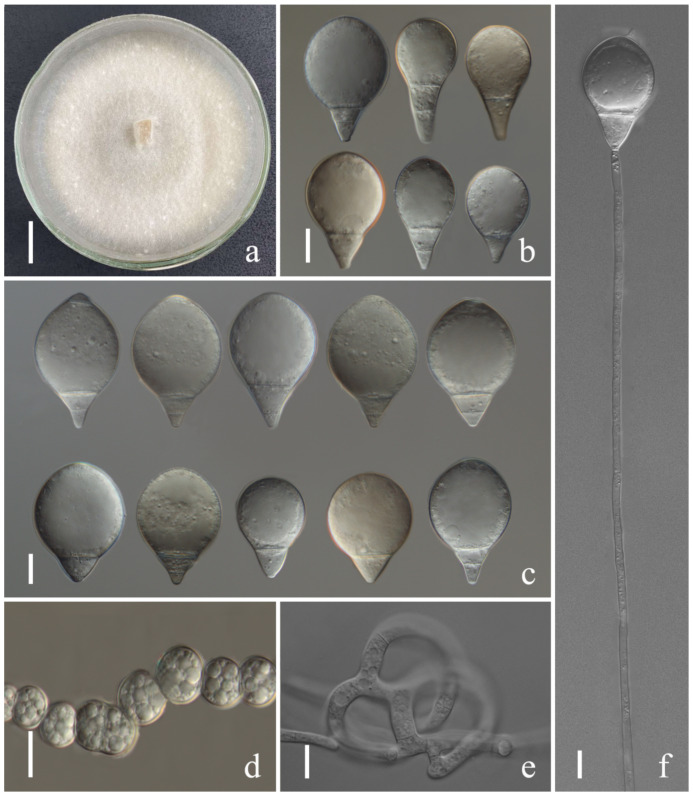
*Arthrobotrys luquanensis* (CGMCC3.20894). (**a**) Colony. (**b**,**c**) Conidia. (**d**) Chlamydospores. (**e**) Trapping device: adhesive networks. (**f**) Conidiophore. Scale bars: (**a**) = 1 cm, (**b**–**f**) = 10 µm.

*Arthrobotrys luquanensis* F. Zhang & X.Y. Yang sp. nov. (Figure 5).

Index Fungorum number: IF 557884; Facesoffungi number: FoF 10763.

Etymology: The species name “luquanensis” refers to the name of the sample collection site: Luquan County, Kunming City, Yunnan Province, China.

Material examined: CHINA, Yunnan Province, Kunming City, Luquan County, 26°10′33.20″ N, 102°45′43.50″ E, from terrestrial soil, 24 May 2017, F. Zhang. Holotype CGMCC3.20894, deposited in the China General Microbiological Culture Collection Center. Ex-type culture DLUCC 19-1, deposited in the Dali University Culture Collection.

*Colonies* on PDA white, cottony, growing rapidly, reaching 55 mm diameter after 10 days in the incubator at 27 °C. *Mycelium* partly superficial, partly immersed, composed of septate, branched, smooth hyphae. *Conidiophores* 216–522 µm (x = 346.5 µm, *n* = 50) long, 2.5–6.5 µm (x = 4.3 µm, *n* = 150) wide at base, gradually tapering upwards to apex, 1.5–3.5 (2.3) µm (x = 2.3 µm, *n* = 50) wide at apex, erect, septate, unbranched, hyaline, bearing a single holoblastic conidium at apex. *Conidia* 28–53.5 × 17–2.5 µm (x = 40.9 × 26.3 μm, *n* = 50), subglobose to widely ovate, with largest cell located at supramedian towards and rounded apex, tapering towards the subacute with truncate at base, 1–2-septate, mostly located at base, sometimes 3-septate (with 2 septa located at basal part and 1 at apex), hyaline, smooth-walled. *Chlamydospores* 6.5–17.5 × 6–14 µm (x = 11.2 × 9.1 μm, *n* = 50), globose to subglobose, ellipsoidal, in chains, hyaline, guttulate, rough-walled. Captures nematodes with adhesive networks.

Additional specimen examined: CHINA, Yunnan Province, Kunming City, Luquan County, 26°10′33.20″ N, 102°45′43.50″ E, from terrestrial soil, 24 May 2017, F. Zhang. Living culture YXY87.

Notes: The phylogenetic analyses revealed that *Arthrobotrys luquanensis* is related to *A. multiformis* and *A. iridis*. *A. luquanensis* was 9.5% (56/590 bp) and 8% (47/589 bp) different from *A. multiformis* and *A. iridis* in ITS sequences, respectively. In morphology, *A. luquanensis* is similar to *A. cookedickinson* and *A. sphaeroides* in simple conidiophores and subfusiform or obovate conidia [4,19,39,40], whereas the conidia of *A. luquanensis* are wider than those of *A. cookedickinson* [*A. luquanensis*, 28.1–53.3 (40.9) × 17–32.4 (26.3) µm versus *A. cookedickinson*, 30–52.5 (42) × 15–22.5 (17.5) µm] and bigger than those of *A. sphaeroides* [*A. luquanensis*, 28.1–53.3 (40.9) × 17–32.4 (26.3) µm versus *A. sphaeroides*, 20–44 (32) × 17–25 (20.4) µm].

**Figure 6:**
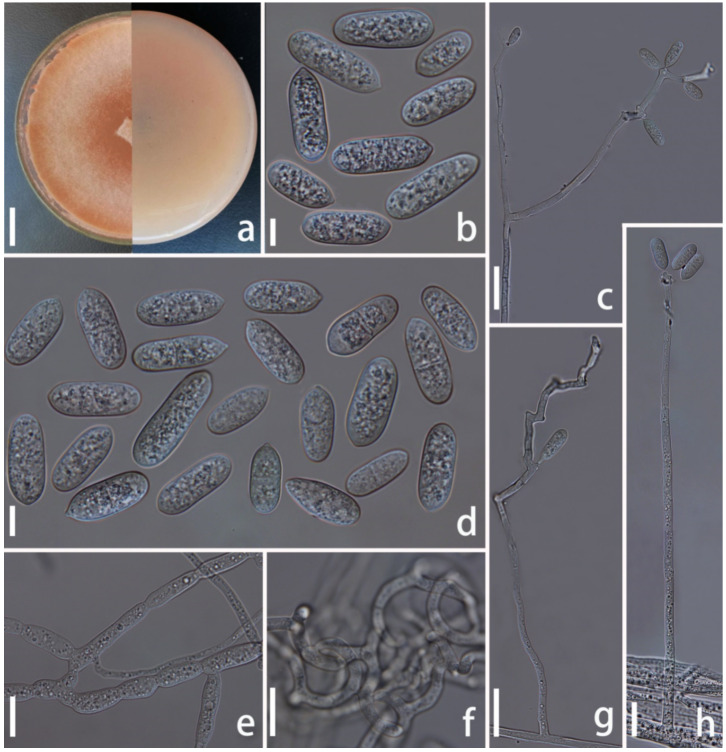
*Arthrobotrys shuifuensis* (CGMCC3.19716). (**a**) Colony. (**b**,**d**) Conidia. (**e**) Chlamydospores. (**c**,**g**,**h**) Conidiophores. (**f**) Trapping device: adhesive networks. Scale bars: (**a**) = 1 cm, (**b**,**d**,**e**) = 10 µm, (**c**,**f**–**h**) = 20 µm.

*Arthrobotrys shuifuensis* F. Zhang & X.Y. Yang sp. nov. (Figure 6).

Index Fungorum number: IF556937; Facesoffungi number: FoF 10764.

Etymology: The species name “shuifuensis” refers to the name of the sample collection site: Shuifu County, Zhaotong City, Yunnan Province, China.

Material examined: CHINA, Yunnan Province, Zhaotong City, Shuifu county, 28°32′31.80″ N, 104°19′9.50″ E, from terrestrial soil, 16 June 2017, F. Zhang. Holotype CGMCC3.19716, deposited in the China General Microbiological Culture Collection Center. Ex-type culture DLUCC 15-1, deposited in the Dali University Culture Collection.

*Colonies* on PDA initially white and turned to pink tinged after 2 weeks, cottony, rapidly growing, reaching 50 mm diameter after 9 days in the incubator at 26 °C. *Mycelium* partly superficial, partly immersed, composed of septate, branched, smooth hyphae. *Conidiophores* 105–305 µm (x = 218.2 µm, *n* = 50) long, 3–5 µm (x = 3.8 µm, *n* = 50) wide at base, gradually tapering upwards to apex, 1.5–3.5 µm (x = 2.5 µm, *n* = 50) wide at apex, erect, septate, unbranched or rarely branched, hyaline, producing several separate nodes by repeated elongation of conidiophores, with each node consisting of 2–8 papilliform bulges and bearing polyblastic conidia. *Conidia* 17–36 × 5–12.5 µm (x = 27.2 × 8.2 μm, *n* = 50), oblong or capsule-shaped, narrower towards the lower and pointed base, 1-septate, median septum, hyaline, rough-walled. *Chlamydospores* 6–18 × 3–7.5µm (x = 9.7 × 8.2 μm, *n* = 50), cylindrical, in chains, hyaline, rough-walled. Capturing nematodes with adhesive networks.

Additional specimen examined: CHINA, Yunnan Province, Zhaotong City, Shuifu County, 28°32′31.80″ N, 104°19′9.50″ E, from terrestrial soil, 16 June 2017, F. Zhang. YXY48.

Notes: Phylogenetic analysis showed that *Arthrobotrys shuifuensis* is the closest species to *A. arthrobotryoides*, there are 9.6% (57/596 bp) differences in ITS sequence between them. Morphologically, this species is similar to *A. arthrobotryoides* in their capsule-shaped, 1-septate conidia, whereas the conidia of *A. shuifuensis* are significantly longer than those of *A. arthrobotryoides* [*A. shuifuensis*, 17–36 (27.2) µm versus *A. arthrobotryoides* 20–22 µm]. In addition, the conidiophores of *A. arthrobotryoides* are unbranched and produces a continuous irregularly swollen node at apex, while the conidiophores of *A. shuifuensis* are branched, producing several separate nodes with the repeated elongation of the conidiophores [19,78].

**Figure 7:**
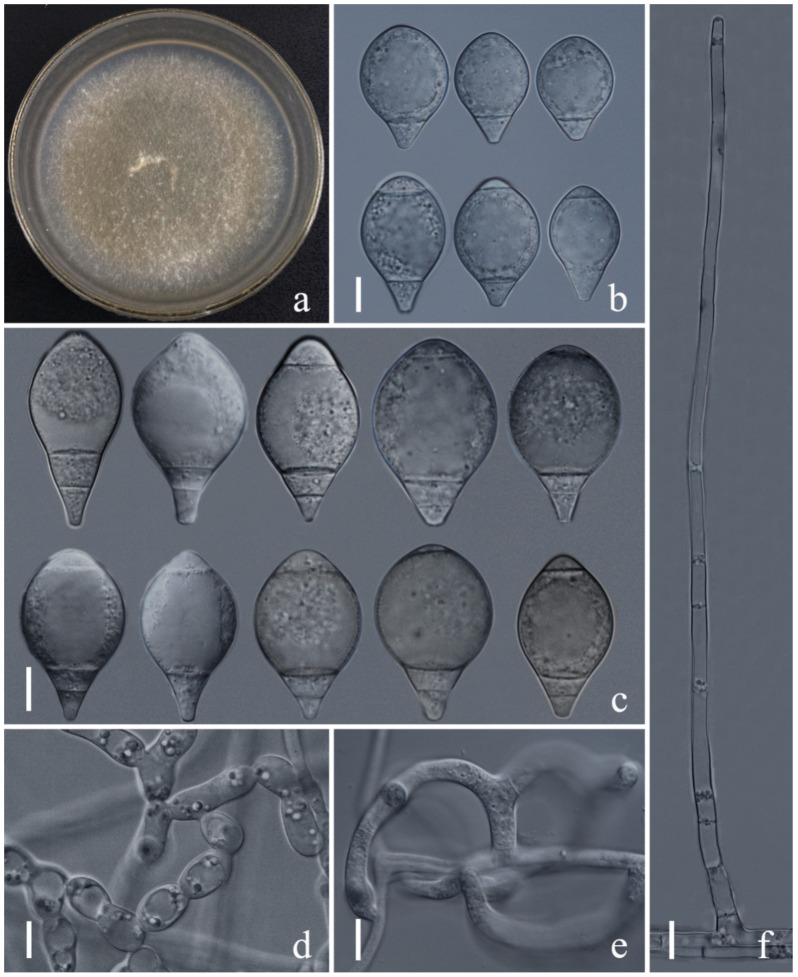
*Arthrobotrys zhaoyangensis* (CGMCC3.20944). (**a**) Colony. (**b**,**c**) Conidia. (**d**) Chlamydospore. (**e**) Trapping device: adhesive network. (**f**) Conidiophores. Scale bars: (**a**) = 1 cm, (**b**–**f**) = 10 µm.

*Arthrobotrys zhaoyangensis* F. Zhang & X.Y. Yang sp. nov. (Figure 7).

Index Fungorum number: IF 556055; Facesoffungi number: FoF 10765.

Etymology: The species name “zhaoyangensis” refers to the name of the sample collection site: Zhaoyang County, Zhaotong City, Yunnan Province, China.

Material examined: CHINA, Yunnan Province, Zhaotong City, Zhaoyang County, 27°29′43.20″ N, 103°10′22.50″ E, from freshwater sediment, 14 April 2015, F. Zhang. Holotype CGMCC3.20944, deposited in the China General Microbiological Culture Collection Center. Ex-type culture DLUCC 20-1, deposited in the Dali University Culture Collection.

*Colonies* on PDA white, cottony, growing rapidly, reaching 48 mm diameter after 10 days in the incubator at 27 °C. *Mycelium* partly superficial, partly immersed, composed of septate, branched, smooth hyphae. *Conidiophores* 207–498 µm (x = 316.5 µm, *n* = 50) long, 3–9.5 µm (x = 5.9 µm, *n* = 50) wide at base, gradually tapering upwards to apex 2–4 µm (x = 2.6 µm, *n* = 50) wide at apex, erect, septate, unbranched, hyaline, bearing a single holoblastic conidium at apex. *Conidia* 25.5–52 × 14–32 µm (x = 35.4 × 22.9 μm, *n* = 50), subglobose, obovoid to obpyriform, wider at median towards supramedian, rounded at apex, tapering towards narrow with subacute and truncate base, 1–3-septate, mostly 3-septate (2 septa at base and 1 at apex), hyaline, rough to smooth-walled. *Chlamydospores* 12.5–31.5 × 6.6–12.5 µm (x = 19.2 × 9.4 μm, *n* = 50) cylindrical, globose or ellipsoidal, in chains, hyaline, guttulate. Captures nematodes with adhesive network.

Additional specimen examined: CHINA, Yunnan Province, Zhaotong City, Zhaoyang County, 27°29′43.20″ N, 103°10′22.50″ E, from freshwater sediment, 14 April 2015, F. Zhang. Living culture YXY86.

Notes: Phylogenetic analysis revealed that the systematic position of *Arthrobotrys zhaoyangensis* is uncertain but showed significant distinction from known species. *A. zhaoyangensis* is most similar to *A. sinensis* and *A. sphaeroides*. *A. zhaoyangensis* can be distinguished from *A. sinensis* and *A. sphaeroides* by bigger conidia [*A. zhaoyangensis*, 25.3–52.1 (35.4) × 14–31.8 (22.9) µm versus *A. sinensis* 23.5–30 (27.6) × 17–25 (20) µm, versus *A. sphaeroides* 20–44(32) × 17–25(20.4) µm]. In addition, these three species differ slightly in the number of septation on conidia; *A. zhaoyangensis* produces 1–3-septate conidia (mostly 3-septate), while the conidia of *A. sinensis* are 2-septate; *A. sphaeroides* sometimes produces aseptate conidia [39,79].

### 3.3. Key to Known Species of Arthrobotrys

1. Conidia 0–1-septate……………………………………………………………………………2
1. Conidia multi-septate…………………………………………………………………………30
2. Conidia mostly aseptate………………………………………………………………………3
2. Conidia mostly 1-septate………………………………………………………………………6
3. Conidiophores branched near apex, producing a node at each branch, or producing several separate nodes by repeated elongation; conidia ovate, with a papilliform bulge at the base………………………………………………………………*A. botryospora*
3. Conidiophores unbranched……………………………………………………………………4
4. Conidiophores with a cluster short denticles at apex; conidia obovoid, 15–31 (23.5) × 10–20 (15.9) μm……………………………………………………………………*A. amerospora*
4. Conidiophores producing several clusters of short denticles by repeated elongation.…5
5. Conidia elongated, ellipsoid–cylindrical, 0–1-septate, mostly non-septate, 17.5–32.5 (22.6) × 2.75–7.5 (5.5) μm………………………………………………………*A. yunnanensis*
5. Conidia elongated, ellipsoidal, non-septate, constricted at the base, 11–16.8 × 5–6.6 μm………………………………………………………………………………*A. nonseptata*
6. Conidia develop on short denticles…………………………………………………………7
6. Conidia develop on nodes.……………………………………………………………………13
7. Conidia curved…………………………………………………………………………………8
7. Conidia straight………………………………………………………………………………10
8. Conidiophores unbranched, conidia in loose capitate arrangement at apex; conidia ellipsoid, mostly curved, 20–47.5 (30.9) × 7–12.5 (10.3) μm…………………*A. musiformis*
8. Conidiophores branched, producing several clusters short denticles by repeated elongation…………………………………………………………………………………………9
9. Conidiophores simple or occasionally branched; conidia elongate-obovoid or elongate-ellipsoidal, 1-septate, straight or curved, 33.5–57 × 11–15.5 µm………*A. shahriari*
9. Conidiophores branched; macroconidia 1-septate, straight or slightly curved, 18–44.5 (28.4) × 5–11.5 (8.7) µm, microconidia aseptate…………………………*A. eryuanensis*
10. Conidiophores producing short denticles by repeated elongation; conidia 1-septate near the base, obpyriform, sometimes constricted at the septum, 24–32.5 × 12.5–20 µm…………………………………………………………………………………*A. perpasta*
10. Conidiophores with clustered short denticles at apex; conidia in loose capitate arrangement at apex…………………………………………………………………………11
11. Conidia clavate, 1-septate at median or submedian, slightly constricted at the septum, 20–37.5 (27.9) × 7.5–10 (8.8) µm…………………………………*A. javanica*
11. Conidia obovoid or obpyriform…………………………………………………………12
12. Conidia obovoid, 1-septate near the base, apical cell much larger, smaller at basal cell, 28.5–32 (30) × 18–20.5 (20) µm…………………………………………………*A. obovata*
12. Conidia obpyriform, 1-septate at submedian, slightly constricted at the septum, 21.4–26.9 × 11.6–15.6 µm……………………………………………*A. koreensis*
13. Conidia develop on short denticles or obscure nodes of conidiophores…………14
13. Conidia develop in clusters on swollen nodes of conidiophores…………………17
14. Conidiophores branched, producing short denticles by repeated elongation; conidia obovate, elongate–obovate, 22.5–32 × 11–22.5 µm…………………………*A. chazarica*
14. Conidiophores unbranched; conidia clavate or pyriform…………………………15
15. Conidia develop on apical conidiophores, conidia clavate, 0 or 1-septate, constricted at the base, 30–45 × 8–11 µm……………………………… *A. pseudoclavata*
15. Conidia pyriform, 1-septate near the basal, apical cell much larger, smaller at basal cell; conidiophores producing several short denticles by repeated elongation………16
16. Conidia perceptibly constricted at the septum, 25–33.8 × 12.5–16.3 µm…*A. paucispora*
16. Conidia non-constricted, 25–35 × 18–24 µm………………………………*A. cystosporia*
17. Conidiophores branched………………………………………………………………18
17. Conidiophores unbranched………………………………………………………………24
18. Conidia 1-septate at median………………………………………………………………19
18. Conidia 1-septate at submedian…………………………………………………………21
19. Conidia elongate–elliptical or cylindrical, 7.5–27.5 (15.8) × 5–10.5 (6.6) µm…*A. superba*
19. Conidia short elliptical to oblong or capsule-shaped……………………………………20
20. Conidiophores occasionally branched, with distinct continuous swollen apical nodes; conidia ellipsoidal, 20–22 × 9–10 µm…………………………………*A. arthrobotryoides*
20. Conidiophores usually branched, bearing conidia on slightly swollen nodes; conidia capsule-shaped, 17–36 (27.2) × 5–12.5 (8.2) µm…………………………*A. shuifuensis*
21. Conidiophores bearing conidia on apical nodes; conidia oblong–pyriform, 20–27.5 (24.4) × 7.5–12.5 (10.8) µm…………………………………………………………*A. robusta*
21. Conidiophores producing several separate nodes by repeated elongation…………22
22. Conidia ellipsoid, elongate–obovate, 10–20 (17.5) × 5–8 (6.2) µm……………*A. cladodes*
22. Conidia obovoid, obpyriform, or ovoid…………………………………………………23
23. Conidia subglobose or elliptical, 14.8–21.5 (18.3) × 10.1–16.3 (13.5) µm…… *A. latispora*
23. Conidia obovoid or obpyriform, 1 septum at submedian, slightly constricted at the septum, 14–26 × 7.5–13 µm………………………………………………*Orbilia jesu-laurae*
24. Condia develop on apical node of conidiophores………………………………………25
24. Conidiophores producing several separate nodes by repeated elongation…………27
25. Conidia non-constricted at the septum, obconical or ellipsoidal, 25–50 × 10–15 µm…………………………………………………………………………………*A. flagrans*
25. Conidia obconical or pyriform, constricted at the septum……………………………26
26. Conidia larger size, constricted at septum, 21–42 (30.5) × 8–15 (12.7) µm……………………………………………………………………………*A. apscheronica*
26. Conidia small size, perceptibly constricted at the septum, 15–37.5 (28.4) × 7.5–14.5 (11.8) µm…………………………………………………………………………*A. conoides*
27. Conidia 1-septate at median……………………………………………………………28
27. Conidia 1-septate at submedian………………………………………………………….29
28. Conidiophores producing continuously expanded node or several separate nodes by repeated elongation; conidia cylindric, long ellipsoid, larger size, 13–22 × 3–7 µm…………………………………………………………………………………*A. anomala*
28. Conidiophores producing several slightly swollen nodes by repeated elongation; conidia ovate, oblong, cylindric, smaller size, 10–20 (14.6) × 2.5–5 (4) µm…*A. dendroides*
29. Conidia obpyriform or drop-shaped, some with a bud-like projection at the base, smaller size, 11.2–26.4(18.6) × 6.6–14.4(10.8) µm………*A. jinpingensis*
29. Conidia pyriform or obovoid, slightly constricted at the septum, larger size, 17–35 (23) × 8.5–16 (12) µm…………………………………………………………*A. oligospora*
30. Conidia without largest cell, with several septa, uniformly distributed among conidial cells ……………………………………………………………………………………31
30. Conidia with largest cell…………………………………………………………………37
31. Y-shaped conidia develop on conidiophores………………………………………*A. iridis*
31. Conidia other type, never Y-shaped…………………………………………………32
32. Conidiophores branched……………………………………………………………….33
32. Conidiophores unbranched………………………………………………………………34
33. Macroconidia spindle-shape or clavate, with 1–7-septate, mostly 2–5, 37.5–100 (70) × 10–17.5 (14.3) µm, microconidia spindle-shape, 0 or 1-septate…………*A. dianchiensis*
33. Conidia elongate–ovate to elongate–doliform or ellipsoidal, with 1–3-septate, 28.5–56 × 11.5–22.5 µm……………………………………………………….……*A. tabrizica*
34. Conidia bearing on apical conidiophores………………………………………………35
34. Conidiophores producing several cluster conidia by repeated elongation…………36
35. Several conidia develop on apical conidiophores, macroconidia elongate-fusiform, clavate, 4–12-septate; microconidia clavate, cylindric–clavate, 0 or 1-septate………………………………………………………………………*A. multiformis*
35. Conidiophores bearing single conidium; conidia clavate, sometimes slightly curved, 2–9-septate, 22.5–73.8 (50.6) × 5–10 (6.6) µm………………………*A. shizishanna*
36. Conidiophores with inconspicuous short denticles; macroconidia fusoid-shaped, curved, 2–4-septate, mostly 3–4, 30–50 (45.1) × 8–16.5 (12.2) µm, microconidia ellispsoid, slightly curved, 1 or 2-septate……………………………………*A. polycephala*
36. Conidiophores producing several short denticles by repeated elongation; conidia elongate–pyriform, 1–3-septate, mostly 2 or 3, 17–38 × 6.5–11.5 µm………*A. pyriformi*
37. Conidiophores branched…………………………………………………………………38
37. Conidiophores unbranched……………………………………………………….….……43
38. Conidiophores bear a single conidium………………………………………………39
38. Conidiophores bear several conidia…………………………………………………40
39. Conidia globose or obpyriform, 1–2-septate, 25–37.5 × 15–22.5 µm……*A. globospora*
39. Conidia subspherical or obovoid or subfusiform, 1–3-septate, 23.5–30 (27.6) × 17–25 (20) µm……………………………………………………………………………*A. sinensis*
40. Conidia in capitate arrangement at apex of conidiophores…………………………41
40. Conidia in non-capitate arrangement on conidiophores……………………………42
41. Conidia obovoid or ellipsoidal, 1–4-septate, mostly 1, 18–36 (28.1) × 12–20 (15.3) µm……………………………………………………………………………*A. azerbaijanica*
41. Conidia pyriform, 1–2-septate, mostly 1, 7.5–22.5 (15.8) × 5–10 (6.6) µm…*A. oviformis*
42. Conidiophores bearing 1 conidium, sometimes 2 conidia; conidia elliptic, top-shaped, 0–2-septate, 17.5–30 (23.2) × 12.5–20 (14.8) µm…………………………………*A. indica*
42. Conidiophores bearing several conidia; macroconidia subfusiform, 2–4-septate, 40–65 (52) × 17–23 (20) µm, microconidia obovoid, aseptate ………*A. oudemansii*
43. Conidiophores bear several conidia ………………………………………………44
43. Conidiophores bear a single conidium ………………………………………………52
44. Conidiophores bear several conidia near apex by repeated elongation…………45
44. Conidiophores bear several conidia at apex…………………47
45. Conidia elongate–ellipsoidal to broadly fusiform, 1–3-septate, mostly1 or 2, 25–50 × 17.5–25 µm……………………………………………………………………*A. vermicola*
45. Conidiophores producing denticles by repeated elongation, conidia fusiform, elongate–fusoid or clavate ……………………………………………………………………46
46. Conidia variable in shape, broadly turbinate to elongate–fusoid, ellipsoidal, fusiform, clavate, 1–3-septate, mostly 2, 25–50 (38.9) × 12–24 (17.3) µm…………*A. mangrovispora*
46. Conidia fusiform, sometimes slightly curved, 1–6-septate, mostly 2–3, 36.6–79.3 (57) × 11–17.5 (14) µm…………………………………………………………………*A. scaphoides*
47. Conidia spindle-shaped, curved,1–4-septate, 25–76 × 7–16 µm………*A. gampsospora*
47. Conidia straight……………………………………………………………………………48
48. Conidia 0–3-septate, mostly 1 or 2………………………………………………………49
48. Conidia 1–4-septate, mostly 3 or 4………………………………………………………50
49. Conidia cymbiform or fusiform, mostly 2-septate, 22.5–45 (27.2) × 10–20 (13.9) µm…………………………………………………………………………*A. microscaphoides*
49. Conidia pyriform, clavate, mostly 1 or 2 septate, 25–40 (17.5) × 7.5–19 (15.4) µm………………………………………………………………………………*A. clavispora*
50. Conidia small, subfusiform, 1–4-septate, mostly 3, 30–60 (36.2) × 15–30(20.2) µm………………………………………………………………………………*A. thaumasia*
50. Conidia larger, ellipsoidal, fusoid–ellipsoidal……………………………………51
51. Conidia ellipsoidal, fusoid–ellipsoidal, 2–4-septate, mostly 4, 46–70 (62.3) × 21–29 (24.7) µm……………………………………………………………………………*A. psychrophila*
51. Conidia fusiform, elongate–ellipsoidal or obovoid, 2–4-septate, mostly 3 or 4, 40–75 × 18–35 µm……………………………………………………………………*A. megalospora*
52. Conidia spindle-shaped, globose, 1–3-septate, mostly 2 or 3, 37–55 (49) × 17.5–35 (28) µm, microconidia ellipsoid, aseptate……………*A. eudermata*
52. Without microconidia……………………………………………………………………53
53. Conidia turbinate or napiform, 1–2-septate, mostly 1 near the base, the largest cell at the apex of conidia, 15–26 (22.5) × 17.5–37.5 (28.5) µm………………………*A. janus*
53. Conidia with more than 2 septa; the largest cell is located in the apex or center of conidia………………………………………………………………………………………54
54. The septum of the conidia is not more than 3…………………………………………55
54. Conidia 1–5-septate………………………………………………………………………58
55. Conidia clavate, obovoid, or subspherical, 0–3-septate……………………………56
55. Conidia fusiform, 2–3-septate……………………………………………………………57
56. Conidia clavate or obovoid, 1–3-septate, mostly 2–3, 30–52.5 (42) × 15–22.5 (17.6) µm………………………*A. cookedickinson*
56. Conidia subspherical or obovoid, 0–3-septate, mostly 2–3, 20–40 (32) × 17–25 (20.4) µm………………………………………………………………………………*A. sphaeroides*
57. Conidia globose or subfusiform, 2–3-septate, 27–47.5 (32.2) × 17.5–27.5 (22) µm………………………………………………………………………………*A. rutgeriense*
57. Conidia spindle-shaped, fusiform or ellipsoidal, 2–3-septate, 32.5–47.5 (41) × 12.5–17.5 (15.5) µm………………………………………………………………………*A. fusiformis*
58. Conidia 1–5-septate, mostly 3 or 4…………………………………………………….59
58. Conidia 1–4-septate, mostly 2 or 3………………………………………………………61
59. Conidia variable in shape, obpyriform, broadly turbinate, subfusiform, elongate-fusoid or clavate, 1–5-septate, 27–72 (55.8) × 14.5–28.5 (21.9) µm…*A. xiangyunensis*
59. Conidia ellipsoid, obpyriform or subfusiform, 2–5-septate, mostly 3 or 4……………60
60. Conidia ellipsoid, fusiform, 50–65 × 20–25 µm…………………………………*A. reticulata*
60. Conidia obpyriform or subfusiform, 40–90 (54) × 15–27.5 (18) µm………… *A. longiphora*
61. Conidia mostly 2-septate……………………………………………………………………62
61. Conidia mostly 3-septate……………………………………………………………………63
62. Conidia obovate, obpyriform or drop-shaped; the distal cell is much smaller, the largest cell usually at the apex, 28–53.5 (40.9) × 17–32.5 (26.3) µm……………… *A. luquanensis*
62. Conidia obpyriform or subfusiform, the largest cell usually at the centre, 30.5–71.5 (52.7) × 18.5–28.5 (23.9) µm……………………………………………*A. guizhouensis*
63. Conidia, subglobose, obovoid to obpyriform, 25.5–52 (35.4) × 14–32 (22.9) µm……………………………………………………………………………*A. zhaoyangensis*
63. Conidia fusiform or ellipsoid…………………………………64
64. Conidia fusiform to ellipsoid, 32.5–52.5 × 12.5–17.5 µm………………………… *A. salina*
64. Conidia mostly subfusiform, 31.1–55.2 (45.4) × 13.5–24.3 (19.7) µm………*A. lanpingensis*

## 4. Discussion

In this phylogenetic analysis, 65 species of nematode-trapping fungi used in this study were clustered into two large clades according to their mechanisms of catching nematodes. Clade I contained species that catch nematodes with adhesive trapping devices (adhesive nets and knobs). Clade II contained species that catch nematodes with active traps (constricting rings). Within clade I, species were clustered into two clades according to their trap types: one clade contained all species that produce adhesive nets, and the other contained those species that produce adhesive knobs. The results were consistent with previous studies [8,15,57,80] and again emphasized the importance of different types of trapping devices in the division of genera among nematode-trapping fungi. At the genus level, the taxonomy of Orbiliaceae nematode-trapping fungi remains an open question, especially in *Arthrobotrys*, which contains the greatest number of species. Morphologically, 61 species of *Arthrobotrys* can be divided into different groups according to the morphologies of their conidiophores and conidia [19]; however, phylogenetic studies have not supported this division; many phylogenetic clades show low support values, and the phylogenetic position of some *Arthrobotrys* species are unclear. The reason for this dilemma is the lack of molecular data for many species, and the existing data cannot provide a stable phylogenetic placement. Therefore, to thoroughly analyse the taxonomy of nematode-trapping fungi, we should use more comprehensive molecular data in future studies.

The emergence of molecular phylogenetic methods has led to unprecedented breakthroughs in the study of fungal taxonomy. Phylogenetic studies based on only a few molecular barcodes cannot provide sufficient and reliable information for the definition of fungal species; therefore, morphological descriptions of each species are still extremely important [81,82]. However, a significant problem facing fungal taxonomy studies is that the description of species is too shallow [83]. This problem is particularly prominent in Orbiliaceae nematode-trapping fungi and is mainly reflected in two aspects. (1) The descriptions of some morphological characteristics are too indistinct. Among six described species in this study, only *A. eryuanensis* and *A. shuifuensis* could be easily distinguished from known species based on their distinct morphological characteristics. The remaining four species required more detailed characteristics (such as the size of conidia) to be identified from known species. When mycologists measure the size of conidia, they are accustomed to uniformly calculating the size data of conidia with different shapes and septate numbers, and the sizes of these conidia usually show significant differences. This causes the size range of conidia to be too extensive for effective comparisons of different species [4,19]. (2) There are too few morphological features that can be used for species identification; although the description of a species includes many features, such as its trap type, conidia, chlamydospores, and hyphae, only the trap type, conidia, and conidiophores can be used for species identification [4,19]. As an increasing number of new species are established, it is difficult to distinguish some similar species based on these three characteristics only. In conclusion, we should screen all potential morphological features in future studies to identify more features with significance for species identification. On the other hand, we should establish a unified standard morphological feature description model to facilitate comparisons between different species.

After the first nematode-trapping fungus was established in 1839 [6], the history of studies on the diversity of nematode-trapping fungi can be divided into three periods. In the nursery period, from 1839 to 1929, due to the limitation of separation methods, only five species were discovered over 90 years. In the rapid development period, from 1931 to 2009, the separation method improved gradually with the contributions of Drechsler et al. [24,25], and nearly 90 species were described over 80 years. From 2010 to 2019, only three species were discovered over 10 years (http://www.speciesfungorum.org (accessed on 6 March 2022)). These data indicated that the excavation of nematode-trapping fungi seems to have reached a plateau, and over time, it is unlikely that many new species will be discovered. However, in recent years, we have investigated nematode-trapping fungi in Yunnan Province and collected 10 new species (four previously published and six reported in this study) [18], which indicates that there are still many nematode-trapping fungi in nature that have not been discovered. Previous studies on the diversity of nematode-trapping fungi have mainly focused on soil habitat, whereas there have been considerably fewer investigations of aquatic nematode-trapping fungi [48,84,85]. However, three of the six new species described in this paper are from freshwater sediment, suggesting that aquatic habitats may also be important sources of nematode-trapping fungi and should not be ignored in future studies.

## Figures and Tables

**Table 1:** The GenBank accession numbers of the isolates included in this study. Ex-type strains are in bold. The newly generated sequences are indicated in blue.

Taxon	Strain Number	GenBank Accession Number			Reference
		ITS	TEF	RPB2	
***Arthrobotrys amerospora***	**CBS 268.83**	**NR 159625**	**—**	**—**	**[59]**
*Arthrobotrys anomala*	YNWS02-5-1	AY773451	AY773393	AY773422	[57]
*Arthrobotrys arthrobotryoides*	CBS 119.54	MH857262	—	—	[59]
*Arthrobotrys arthrobotryoides*	AOAC	MF926580	—	—	Unpublished
*****Arthrobotrys botryospora*****	**CBS 321.83**	**NR 159626**	**—**	**—**	**[59]**
*Arthrobotrys cladodes*	1.03514	MH179793	MH179616	MH179893	Unpublished
*****Arthrobotrys clavispora*****	**CBS 545.63**	**MH858353**	**—**	**—**	**[59]**
*Arthrobotrys conoides*	670	AY773455	AY773397	AY773426	[57]
*Arthrobotrys cookedickinson*	YMF1.00024	MF948393	MF948550	MF948474	[4]
*Arthrobotrys cystosporia*	CBS 439.54	MH857384	—	—	[59]
*Arthrobotrys dendroides*	YMF1.00010	MF948388	MF948545	MF948469	[4]
*Arthrobotrys dianchiensis*	1.00571	MH179720	—	MH179826	[60]
*Arthrobotrys elegans*	1.00027	MH179688	—	MH179797	Unpublished
*****Arthrobotrys eryuanensis*****	**CGMCC3.19715**	**MT612105**	**OM850307**	**OM850301**	**This study**
*****Arthrobotrys eryuanensis*****	**YXY45**	**ON808616**	**ON809547**	**ON809553**	**This study**
*Arthrobotrys eudermata*	SDT24	AY773465	AY773407	AY773436	[57]
*Arthrobotrys flagrans*	1.01471	MH179741	MH179583	MH179845	Unpublished
*Arthrobotrys gampsospora*	CBS 127.83	U51960	—	—	[61]
*Arthrobotrys globospora*	1.00537	MH179706	MH179562	MH179814	Unpublished
*Arthrobotrys guizhouensis*	YMF1.00014	MF948390	MF948547	MF948471	[4]
*Arthrobotrys indica*	YMF1.01845	KT932086	—	—	[62]
*Arthrobotrys iridis*	521	AY773452	AY773394	AY773423	[57]
*Arthrobotrys janus*	Jan-85	AY773459	AY773401	AY773430	[57]
*Arthrobotrys javanica*	105	EU977514	—	—	Unpublished
*****Arthrobotrys jinpingensis*****	**CGMCC3.20896**	**OM855569**	**OM850311**	**OM850305**	**This study**
*****Arthrobotrys jinpingensis*****	**YXY101**	**ON808621**	**ON809552**	**ON809558**	**This study**
*Arthrobotrys koreensis*	C45	JF304780	—	—	[63]
*****Arthrobotrys lanpingensis*****	**CGMCC3.20998**	**OM855566**	**OM850308**	**OM850302**	**This study**
*****Arthrobotrys lanpingensis*****	**YXY80**	**ON808618**	**ON809549**	**ON809555**	**This study**
*Arthrobotrys latispora*	H.B. 8952	MK493125	—	—	Unpublished
*Arthrobotrys longiphora*	1.00538	MH179707	—	MH179815	Unpublished
*****Arthrobotrys luquanensis*****	**CGMCC3.20894**	**OM855567**	**OM850309**	**OM850303**	**This study**
*****Arthrobotrys luquanensis*****	**YXY87**	**ON808619**	**ON809550**	**ON809556**	**This study**
*Arthrobotrys mangrovispora*	MGDW17	EU573354	—	—	[64]
*Arthrobotrys megalospora*	TWF800	MN013995	—	—	Unpublished
*Arthrobotrys microscaphoides*	YMF1.00028	MF948395	MF948552	MF948476	[4]
*****Arthrobotrys multiformis*****	**CBS 773.84**	**MH861834**	**—**	**—**	**[59]**
*Arthrobotrys musiformis*	SQ77-1	AY773469	AY773411	AY773440	[57]
*Arthrobotrys musiformis*	1.03481	MH179783	MH179607	MH179883	Unpublished
*****Arthrobotrys nonseptata*****	**YMF1.01852**	**FJ185261**	**—**	**—**	**[62]**
*****Arthrobotrys obovata*****	**YMF1.00011**	**MF948389**	**MF948546**	**MF948470**	**[4]**
*Arthrobotrys oligospora*	920	AY773462	AY773404	AY773433	[57]
*Arthrobotrys paucispora*	ATCC 96704	EF445991	—	—	[57]
*Arthrobotrys polycephala*	1.01888	MH179760	MH179592	MH179862	Unpublished
*Arthrobotrys pseudoclavata*	1130	AY773446	AY773388	AY773417	[57]
*Arthrobotrys psychrophila*	1.01412	MH179727	MH179578	MH179832	Unpublished
*Arthrobotrys pyriformis*	YNWS02-3-1	AY773450	AY773392	AY773421	[57]
*Arthrobotrys reticulata*	CBS 550.63	MH858355	—	—	[59]
*Arthrobotrys robusta*	nefuA4	MZ326655	—	—	Unpublished
*Arthrobotrys salina*	SF 0459	KP036623	—	—	Unpublished
*Arthrobotrys scaphoides*	1.01442	MH179732	MH179580	MH179836	Unpublished
*Arthrobotrys shizishanna*	YMF1.00022	MF948392	MF948549	MF948473	[4]
*****Arthrobotrys shuifuensis*****	**CGMCC3.19716**	**MT612334**	**OM850306**	**OM850300**	**This study**
*****Arthrobotrys shuifuensis*****	**YXY48**	**ON808617**	**ON809548**	**ON809554**	**This study**
*Arthrobotrys sinensis*	105-1	AY773445	AY773387	AY773416	[57]
*Arthrobotrys sphaeroides*	1.0141	MH179726	MH179577	MH179831	Unpublished
*Arthrobotrys superba*	127	EU977558	—	—	Unpublished
*Arthrobotrys thaumasia*	917	AY773461	AY773403	AY773432	[57]
*Arthrobotrys vermicola*	629	AY773454	AY773396	AY773425	[57]
*****Arthrobotrys xiangyunensis*****	**YXY10-1**	**MK537299**	**—**	**—**	[17]
*****Arthrobotrys yunnanensis*****	**AFTOL-ID 906**	**DQ491512**	**—**	**—**	**Unpublished**
*** **Arthrobotrys zhaoyangensis** ***	**CGMCC3.20944**	**OM855568**	**OM850310**	**OM850304**	**This study**
*** **Arthrobotrys zhaoyangensis** ***	**YXY86**	**ON808620**	**ON809551**	**ON809557**	**This study**
*Dactylaria higginsii*	CBS 121934	KM009164	—	—	Unpublished
*****Dactylellina appendiculata*****	**CBS 206.64**	**AF106531**	**DQ358227**	**DQ358229**	**[59]**
*****Dactylellina copepodii*****	**CBS 487.90**	**U51964**	**DQ999835**	**DQ999816**	**[61]**
*****Dactylellina mammillata*****	**CBS229.54**	**AY902794**	**DQ999843**	**DQ999817**	**[65]**
*****Datylellina yushanensis*****	**CGMCC3.19713**	**MK372061**	**MN915113**	**MN915112**	**[18]**
*Drchslerella coelobrocha*	FWY03-25-1	AY773464	AY773406	AY773435	[57]
*Drchslerella dactyloides*	expo-5	AY773463	AY773405	AY773434	[57]
*Drchslerella stenobrocha*	YNWS02-9-1	AY773460	AY773402	AY773431	[57]
*Drechslerella brochopaga*	701	AY773456	AY773398	AY773427	[57]
*****Orbilia jesu-laurae*****	**LQ59a**	**MN816816**	**—**	**—**	**[66]**
*Vermispora fusarina*	YXJ02-13-5	AY773447	AY773389	AY773418	[57]

## Data Availability

The data that support the finding of this study are contained within the article.
